# The potential role of sleep quality in the relationship between glymphatic function and migraine frequency: Insights from a cross‐sectional study

**DOI:** 10.1111/head.15019

**Published:** 2025-08-19

**Authors:** Raffaele Ornello, Federico Salfi, Maria Grazia Vittorini, Antonio Innocenzi, Federico De Santis, Federico Bruno, Francesca Pistoia, Michele Ferrara, Alessandra Splendiani, Simona Sacco

**Affiliations:** ^1^ Department of Biotechnological and Applied Clinical Sciences University of L'Aquila L'Aquila Italy

**Keywords:** diffusion tensor imaging along the perivascular space, migraine, migraine comorbidities, sleep

## Abstract

**Objective:**

This study aimed to explore the association between glymphatic function, as assessed by the diffusion tensor imaging along perivascular space (DTI‐ALPS) index, and headache frequency in individuals with migraine. Additionally, it evaluated whether sleep quality modulates this relationship.

**Background:**

Migraine has a complex pathophysiology involving genetic predispositions, comorbidities, and psychosocial factors. Emerging evidence highlights the glymphatic system—responsible for brain waste clearance—as a potential contributor to migraine pathogenesis. As poor sleep quality exacerbates glymphatic dysfunction, it might have an impact on migraine chronification.

**Methods:**

This cross‐sectional study included 106 individuals with migraine (80.2% female; median age: 45.0 years, interquartile range = 37.0–52.0) between June 2018 and February 2020. Glymphatic function was assessed using the DTI‐ALPS index derived from brain magnetic resonance imaging, whereas sleep quality was evaluated with the Pittsburgh Sleep Quality Index. First, we evaluated the association between DTI‐ALPS index and monthly headache days. A second model included the dichotomized Pittsburgh Sleep Quality Index score (“poor” vs. “good” sleepers) and its interaction with the DTI‐ALPS index to examine the moderating role of sleep quality in the relationship between glymphatic function and monthly headache days.

**Results:**

Higher DTI‐ALPS index values were associated with fewer monthly headache days in the overall sample while controlling for age and sex (adjusted Incidence Rate Ratio [IRR] = 0.37; 95% confidence interval [CI] = 0.16–0.86, *p* = 0.020). Additionally, there was a significant interaction between DTI‐ALPS index and sleep quality (adjusted IRR = 0.13; 95% CI = 0.02–0.76, *p* = 0.024) in predicting monthly headache days. We found an association between lower DTI‐ALPS and higher headache days only in participants with poor sleep quality (adjusted IRR = 0.21; 95% CI = 0.08–0.59, *p* = 0.003) whereas no association was found in good sleepers (adjusted IRR = 1.66; 95% CI = 0.38–7.16, *p* = 0.500). Results remained consistent after adjusting for clinical variables such as disease duration, medication overuse, cutaneous allodynia, aura status, and migraine subtype.

**Conclusion:**

Poor sleep quality moderates the association between glymphatic dysfunction and monthly headache days. The results highlight the potential importance of sleep interventions in managing migraine and improving brain glymphatic function.

## Plain Language Summary

People with migraine often report poor sleep, but it is still unclear if and how sleep quality affects the relationship between the glymphatic system (the system that clears waste products from the brain) and migraine symptoms. In this study, we used advanced brain imaging to indirectly measure glymphatic function and examined how it relates to headache frequency and sleep quality. We found that lower glymphatic function was linked to more frequent headaches only in people with poorer sleep quality, suggesting that sleep quality may amplify the impact of brain clearance problems in migraine.

**Abbreviations**
CGRP: calcitonin gene‐related peptide
CSD: cortical spreading depression
DTI‐ALPS: diffusion tensor imaging along the perivascular space
IQR: interquartile range
MRI: magnetic resonance imaging
NB: negative binomial
PSQI: Pittsburgh Sleep Quality Index
ROI: region of interest

## INTRODUCTION

Migraine stands as one of the most prevalent disorders globally, affecting 15.2% of the population and ranking among the foremost causes of disability.^1^, ^2^ Beyond its clinical manifestations, it is now recognized as a complex condition encompassing cognitive complaints, psychiatric disorders, and emotional distress.^2^, ^3^ This relative burden is further compounded by healthcare disparities and underdiagnoses in some countries that limit access to proper care for many patients and contribute to patients' reduced quality of life.^4^

Despite its significant impact, the precise pathophysiology of this condition remains still elusive. Currently, migraine is recognized as a multifaceted disorder, in which an inherent predisposition interacts with comorbidities and psychosocial factors, potentially influencing its course of exacerbation or amelioration.^5^

The recently identified glymphatic system—a network of lymphatic vessels responsible for brain waste clearance and maintaining cerebrospinal fluid homeostasis^6^—has been increasingly implicated in migraine pathophysiology. Possible pathogenetic mechanisms of migraine involving the glymphatic system dysfunction included neuroinflammation, cortical spreading depression (CSD), and calcitonin gene‐related peptide (CGRP) dysregulation.^7^

Clinical studies have sought a relationship between glymphatic function and the presence or severity of migraine with inconsistent results. Some studies found no difference in glymphatic function between individuals with migraine—either episodic or chronic—and healthy controls,^8^, ^9^, ^10^ whereas the comparisons between individuals with chronic migraine and healthy controls showed either a decreased^11^ or an increased glymphatic function.^12^ Notably, all those studies assessed the glymphatic function via the diffusion tensor imaging along the perivascular space (DTI‐ALPS) index, which is a non‐invasive and non‐contrast magnetic resonance imaging (MRI) technique evaluating the glymphatic function by analyzing the interstitial fluid–cerebrospinal fluid diffusion into the perivascular spaces.^13^

One key moderator that could clarify these mixed findings about migraine and glymphatic system is sleep quality. Migraine and sleep disturbances share a complex, bidirectional relationship, with sleep disturbances implicated in both triggering migraine and promoting its chronification.^14^, ^15^, ^16^ The glymphatic system is highly active during slow‐wave sleep, when perivascular channels open to clear brain waste.^17^ Poor sleep quality may disrupt this process, reducing glymphatic flow and potentially exacerbating migraine frequency and severity.^18^ Thus, sleep disturbances might not only influence migraine pathogenesis directly but also modulate the association between glymphatic function and migraine, amplifying its impact on individuals with poor sleep.

The current study aimed to address this gap by examining how sleep quality interacts with glymphatic function in individuals with migraine. First, we evaluated a potential relationship between DTI‐ALPS index and headache frequency, hypothesizing that lower DTI‐ALPS index was associated with more frequent headaches. Second, we hypothesized a moderation role of sleep quality in the glymphatic function‐migraine frequency relationship. Specifically, we expected that individuals with poor sleep quality may show the consequences of glymphatic dysfunction with having more headache days. By exploring this moderating role of sleep, the study seeks to provide a clearer understanding of how these interconnected systems contribute to migraine pathophysiology and chronification, potentially identifying novel avenues for targeted intervention.

## METHODS

### Participants

This observational cross‐sectional study encompassed a consecutive convenience sample of individuals with migraine who were seeking care at a single headache center. The study was approved by the internal review board of the University of L'Aquila (number 05/2018) and participants signed a written informed consent. Methods of the study have been previously published^19^ together with results referring the association between glymphatic system dysfunction and white matter hyperintensities.

The study population consisted of adult men and women diagnosed with migraine with or without aura, episodic or chronic, as per the *International Classification of Headache Disorders, 3rd Edition*.^20^ Inclusion occurred between June 2018 and February 2020 according to the additional following criteria: absence of ongoing migraine preventive medication, absence of severe medical/psychiatric conditions in the medical history, and absence of neurological diseases other than migraine. Individuals with non‐disabling comorbidities were eligible for inclusion at the discretion of the treating physician. Exclusion criteria encompassed the presence of vascular risk factors (e.g., arterial hypertension or diabetes mellitus), medical history of autoimmune diseases potentially linked to white matter damage, brain lesions evident on brain MRI, and signs indicative of cerebral small vessel disease, such as subcortical infarctions, lacunes, or microbleeds.^21^ Although enlarged perivascular spaces could represent a consequence of glymphatic system dysfunction,^22^, ^23^ individuals with enlarged perivascular spaces at brain MRI were excluded to avoid confounding with signs of brain small vessel disease.^24^

Participants' sex, age, duration of migraine (in years), presence of aura or cutaneous allodynia, and monthly headache days over the past 3 months were collected during routine clinical interviews. All clinical information underwent review by two neurologists with extensive experience in headache medicine (R.O., S.S.).

### Brain MRI

**FIGURE 1:**
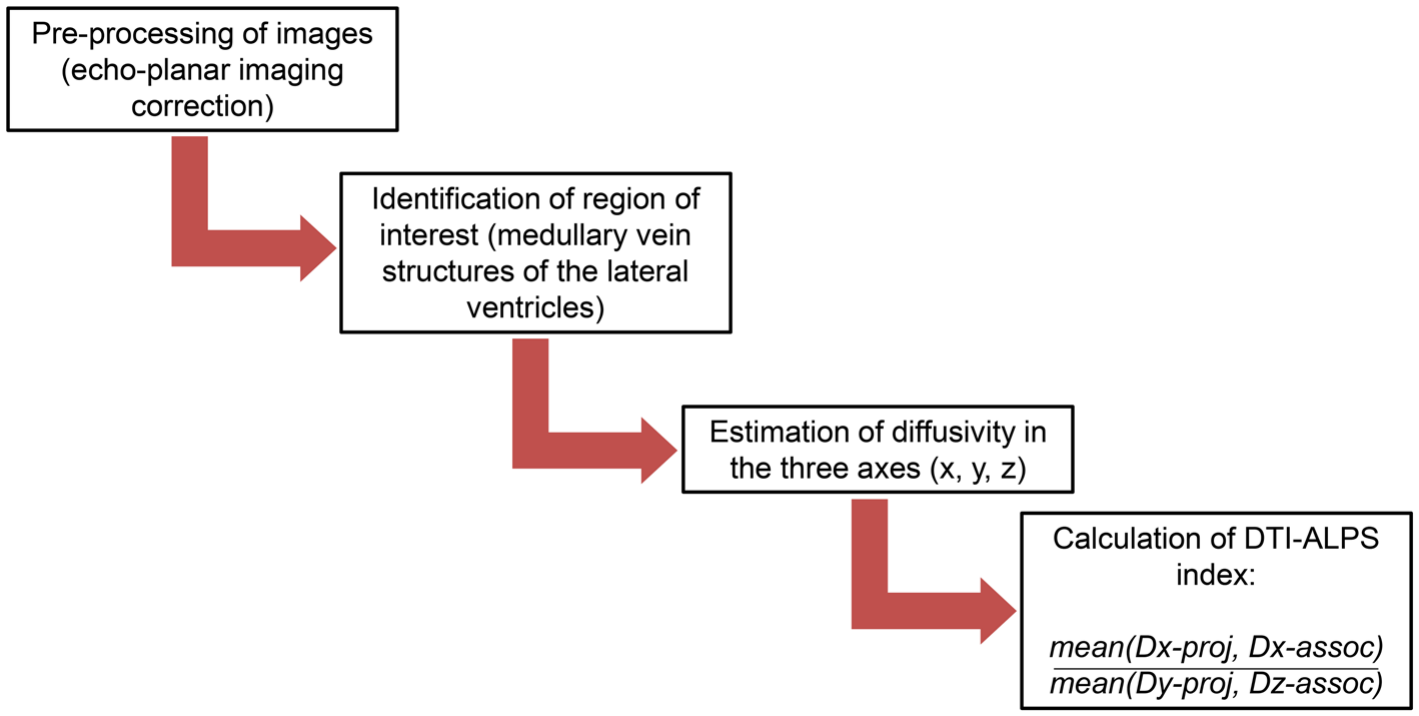
Flowchart for the calculation of diffusion tensor imaging along the perivascular space (DTI‐ALPS). [Color figure can be viewed at wileyonlinelibrary.com]

All brain MRIs were performed during the daytime using the same scanner from the same two operators with specific expertise in neuroradiology (A.S., F.B.). A 3‐Tesla scanner with a 32‐channel head coil (MR750w, GE Healthcare) was used. Individuals all underwent the same brain MRI acquisition protocol, which has been described in a previous article.^19^ All the individuals were headache‐free for ≥24 h from MRI scans. To obtain the ALPS index, we used a previously described method.^13^, ^25^ We processed DTI data using the software DSI‐studio (https://dsi‐studio.labsolver.org), “Chen” version. The software is freely available and was different from that used in a previous publication^19^; hence, DTI‐ALPS index values may vary compared with our previous publication. Echo‐planar imaging correction tool for distortion correction was applied before image analysis. Spherical regions of interest (ROI; 5 mm^2^) were positioned at the level of the projection fibers and the association fibers, concentrically along the medullary vein structures at the level of the lateral ventricles. In each participant, diffusivity in the directions of the x‐axis, y‐axis, and z‐axis of each area was recorded. The DTI‐ALPS index was calculated using the formula: mean(Dx‐proj, Dx‐assoc)/mean(Dy‐proj, Dz‐assoc), where Dx‐proj represents diffusivity along the *x*‐axis in projection fibers, Dx‐assoc diffusivity along the x‐axis in association fibers, Dy‐proj diffusivity along the y‐axis in projection fibers, and Dz‐assoc diffusivity along the z‐axis in association fibers. A flowchart for DTI‐ALPS calculation is reported in Figure 1.

### Assessment of sleep quality

To assess the quality of sleep in our population, we used the Italian version of the Pittsburgh Sleep Quality Index (PSQI),^26^ a self‐report questionnaire that evaluates the quality of sleep over a 1‐month time interval. It includes seven sections (subjective sleep quality, sleep latency, sleep duration, habitual sleep efficiency, sleep disturbances, use of sleeping medication, and daytime dysfunction) with a total amount of 24 items, 19 of which are self‐reported and five of which requires secondary feedback from a room partner^27^; each section is rated from 0 to 3, for a global score of 21. A PSQI score ≤5 refers to people free from sleep disturbances (“good” sleepers), whereas a PSQI score >5 is associated with mild to severe sleep disturbances (“poor” sleepers). PSQI score >5 yields a diagnostic sensitivity of 89.6% and specificity of 86.5% in distinguishing good and poor sleepers.^28^

### Statistical analysis

Our study is a secondary analysis of the collected data and was based on a convenience sample without any formal sample size calculation.

Normality of continuous variables was assessed visually and statistically using the Shapiro–Wilk test. All continuous variables violated the assumption of normality and were therefore summarized using medians and interquartile ranges (IQRs). Headache frequency, treated as a count variable, was likewise reported using median and IQR due to its skewed distribution. Categorical variables were summarized using counts and percentages.

Pearson's correlation was performed to test the association between age and DTI‐ALPS index. Moreover, we employed a negative binomial (NB) regression model to investigate the relationship between the DTI‐ALPS index and headache frequency, considering the number of monthly headache days as the dependent variable and the DTI‐ALPS index as the primary predictor. The statistical approach has been chosen considering the nature of the dependent variable (count data),^29^, ^30^ after excluding the potentiality of using Poisson regression models^31^, ^32^ due to a failure to meet the equidispersion assumption (χ^2^/df = 5.40, exceeding the acceptable threshold of 1.20).^33^ This initial analysis did not include sleep quality to establish the direct predictive value of the DTI‐ALPS index on monthly headache days, while controlling for age and sex, which are known to influence DTI‐ALPS values.^34^ Because this analysis was limited to a single, pre‐specified association, we did not apply correction for multiple testing.

Subsequently, we expanded the model to evaluate the moderating role of sleep quality in this association. Specifically, the NB model included the number of monthly headache days as the dependent variable and incorporated the DTI‐ALPS index, the dichotomized PSQI scores (“good” sleepers vs. “poor” sleepers, based on a cutoff value of 5^30^), and their interaction term as predictors. Age and sex were again included as covariates. We examined model assumptions by checking for overdispersion, linearity in the log scale, residual autocorrelation, and potential collinearity. In both the NB model with and without the DTI‐ALPS × PSQI interaction, these assumptions were satisfied, and goodness‐of‐fit measures further supported our final specification.

Finally, a supplementary model was performed by adjusting for a set of clinically relevant variables (see Table S1). Specifically, we added the years of disease history, medication overuse (presence/absence), cutaneous allodynia (presence/absence), aura status (presence/absence), and migraine type (binary: chronic/episodic) as predictors. These variables were selected based on their potential to act as confounders in the relationship between DTI‐ALPS and migraine frequency.

For all models, two‐tailed statistical significance was set for *p* < 0.050. Statistical analyses were performed using R version 4.1.2 (R Foundation for Statistical Computing, Vienna, Austria) and NB models were fitted using the “GAMLj” (version 3.0.0) R module.

## RESULTS

Among the 147 individuals originally included in our sample,^19^ 106 (72.1%) had available PSQI scores and thus were included in the present analysis. The remaining 41 participants were excluded due to missing PSQI scores. No other missing data were present for the variables included in the analysis.

Among the 106 included individuals, 85 (80.2%) were females, and the median age was 45.0 (Interquartile Range [IQR] , 37.0–52.0) years and the median history of migraine was 22.0 (IQR, 14.0–31.5) years. A total of 74 individuals (69.8%) had a PSQI score >5 (“poor” sleepers) (Table 1).

**TABLE 1:** Characteristics of the study population (*n* = 106).

Variable	
Female, *n* (%)	85 (80.2)
Age years, median (IQR)	45.0 (37.0–52.0)
Chronic migraine, *n* (%)	43 (40.6)
Aura, *n* (%)	30 (28.3)
Cutaneous allodynia, *n* (%)	34 (32.1)
Medication overuse, *n* (%)	28 (26.4)
Monthly headache days, median (IQR)	8 (4.0–14.0)
Years of disease history, median (IQR)	22.0 (14.0–31.5)
PSQI score, median (IQR)	7.0 (5.0–10.0)
PSQI score >5 (poor sleepers), *n* (%)	74 (69.8)

Abbreviations: IQR, interquartile range; PSQI, Pittsburgh Sleep Quality Index.

Pearson's correlation indicated a negative relationship between age and DTI‐ALPS index (*r* = −0.27, *p* = 0.005), with older participants that showed the higher glymphatic dysfunction.

**FIGURE 2:**
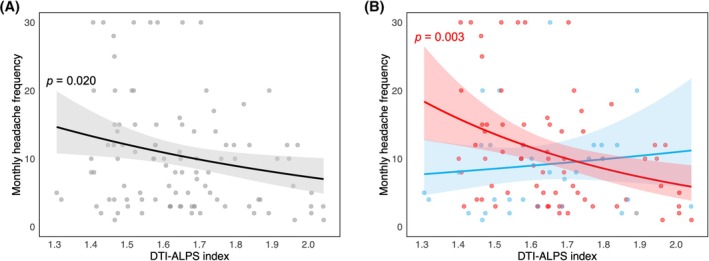
(A) Scatterplot and regression line showing the relationship between diffusion tensor imaging along the perivascular space (DTI‐ALPS) index and monthly headache frequency across the whole sample. (B) Illustration of the relationship between DTI‐ALPS index and monthly headache frequency by sleep quality (poor sleepers: Pittsburgh Sleep Quality Index [PSQI] ≤5, good sleepers: PSQI >5), revealing a significant interaction. Shaded areas represent 95% confidence intervals, whereas *p*‐value is reported for significant associations. [Color figure can be viewed at wileyonlinelibrary.com]

The first NB model revealed a significant negative association between DTI‐ALPS index and monthly headache days while controlling for age and sex (adjusted Incidence Rate Ratio [IRR] = 0.37; 95% Confidence Interval [CI] = 0.16–0.86, *p* = 0.020). Lower glymphatic function (as indicated by lower DTI‐ALPS index) was associated with higher number of monthly headache days (Figure 2A). On a practical note, the model suggested that a one standard deviation decrease in DTI‐ALPS index (−0.175 units) was associated with an estimated 19.0% increase in monthly headache frequency in the overall sample. Age was not a significant predictor of monthly headache days (adjusted IRR = 1.01; 95% CI = 0.99–1.03, *p* = 0.168), and female patients reported a higher number of monthly headache days (adjusted IRR = 1.46; 95% CI = 1.00–2.11, *p* = 0.049).

Results from the second NB model indicated a significant interaction between DTI‐ALPS index and sleep quality (adjusted IRR = 0.13; 95% CI = 0.02–0.76, *p* = 0.024) in relation to monthly headache days while adjusting for age and sex. This outcome suggested a different relationship between DTI‐ALPS index and monthly headache days according to sleep quality. Indeed, simple slope analyses showed that lower DTI‐ALPS index was significantly related with a higher number of monthly headache days in poor sleepers (adjusted IRR = 0.21; 95% CI = 0.08–0.59, *p* = 0.003), translating into an estimated 38.8% increase in monthly headache days for each one standard deviation decrease in DTI‐ALPS index (−0.180); however, the same relationship was not present in individuals with good sleep quality (adjusted IRR = 1.66; 95% CI = 0.38–7.16, *p* = 0.500) (Figure 2B).

The supplementary model (Table S1) adjusting for a set of clinically relevant variables (years of disease history, medication overuse, cutaneous allodynia, aura status, and migraine type) confirmed the significant moderation role of sleep quality on the relationship between DTI‐ALPS index and monthly headache days (adjusted IRR = 0.20; 95% CI = 0.05–0.89, *p* = 0.03).

## DISCUSSION

In the last decade, there has been a growing interest in the role of the glymphatic system in the pathogenesis of migraine.^7^ In this study, we investigated the association between glymphatic function and monthly headache days in a cohort of 106 patients with migraine, examining the potential moderating role of sleep quality. Our findings suggest that reduced glymphatic function, as measured by the DTI‐ALPS index, is associated with a higher number of monthly headache days. However, this relationship is significantly influenced by sleep quality, with increased monthly headache days associated with higher glymphatic dysfunction only in poor sleepers.

The negative correlation between DTI‐ALPS index and headache frequency aligns with previous evidence implicating glymphatic dysfunction in migraine.^35^ The glymphatic system is responsible for clearing brain waste, including pro‐inflammatory cytokines and neuropeptides such as CGRP, which are known contributors to migraine pathogenesis.^7^ The impaired glymphatic function could exacerbate these mechanisms, promoting neuroinflammation and cortical spreading depolarization phenomena, both of which are central to migraine development and chronification.^36^

Our results also provide new insights into the interaction between sleep quality and glymphatic function in migraine. During slow‐wave sleep, glymphatic activity peaks^17^ and poor sleep quality has been linked to reduced glymphatic clearance.^37^, ^38^ This disruption may amplify the accumulation of neurotoxic waste products, exacerbating migraine frequency in individuals with poor sleep.^18^ On the other hand, no significant association was observed between glymphatic function and headache frequency in good sleepers, suggesting that adequate sleep may protect against the impact of glymphatic dysfunction on migraine. Moreover, we showed a consistent age‐related decline in DTI‐ALPS values in our sample, which is consistent with recent reports on patients with migraine^35^, ^39^ and larger investigations on the UK Biobank participants.^40^

Our findings could contribute to clarifying the complex relationship between the glymphatic system and migraine, providing a potential interpretative key for the inconsistencies observed in the literature.^8^, ^12^ Indeed, it should be acknowledged that our findings are inconsistent with two studies demonstrating no glymphatic alterations in patients with migraine^8^ or even enhanced glymphatic functionality compared to healthy controls.^12^ Notably, these discrepancies may not be ascribed to methodological issues because all the aforementioned human studies used the same metric from MRI (the DTI‐ALPS index) as an indirect estimate of glymphatic functioning. The moderating role of sleep quality identified in this study suggests that assessing sleep disturbances might be important in future research investigating the role of the glymphatic system in migraine.

Although the DTI‐ALPS index provides a valuable noninvasive measure of glymphatic activity in clinical settings,^41^ a review highlighted that the ALPS method should be interpreted cautiously.^42^ As discussed, a lower DTI‐ALPS index does not necessarily correspond to a definitive marker glymphatic dysfunction but rather indicates that water diffusivity in the perivascular direction is reduced under certain conditions. This caution is reinforced by the fact that the ALPS index is an indirect biomarker of glymphatic activity as it is influenced by multiple factors, including white matter fiber geometry, partial volume effects, and overall anatomical variability. Moreover, alternative noninvasive imaging modalities, such as contrast‐based tracer methods, perivascular space volumetry, or advanced diffusion/Arterial Spin Labeling‐based approaches, may offer complementary and potentially more direct insights into brain interstitial fluid dynamics (see Table 2 for an overview). Because no single imaging modality can fully assess the complexity of glymphatic flow, future investigations would benefit from combining multiple techniques to obtain a more comprehensive evaluation of glymphatic function.

**TABLE 2:** Main features of different techniques to analyze the glymphatic system.

Technique	Pros	Cons
CE‐MRI	Non‐invasive technique Whole brain images 3D visualization	Low spatial and temporal resolution Movement artifacts
DTI‐ALPS	Quantify the diffusion of water molecules within the brain's interstitial space, providing valuable insights into glymphatic function	Low specificity
Arterial spin labeling Chemical exchange saturation transfer Intravoxel incoherent motion	Blood brain barrier permeability Assess of solutes concentration at two orders of magnitude lower than traditional MRI Diffusion/perfusion effect evaluation of blood motion	Indirect measurements of the glymphatic system function Need of integration with other techniques
Ultra‐high MRI	Better detection of PVS abnormalities	Movement artifacts dishomogeneity in magnetic field Difficulty to identify subcortical PVS radiofrequency absorption rate Lower compatibility of medical devices
Positron emission tomography	Use of radiolabeled tracers Whole brain images Quantify the glymphatic system clearance	Low spatial resolution Movement artifacts
Transcranial Doppler ultrasound	Study glymphatic pulsations and CSF dynamics in humans	Low specificity

Abbreviations: (CE)‐MRI, (contrast‐enhanced) magnetic resonance imaging; CSF, cerebrospinal fluid; DTI‐ALPS, diffusion tensor imaging along the perivascular space; PVS, perivascular space.

To our knowledge, this investigation is the first to address the relationship between glymphatic system function and sleep quality in individuals with migraine. We employed a globally recognized and validated scale to evaluate sleep quality in individuals with migraine, along with advanced MRI software to assess glymphatic system function. Besides, our sample size was larger than that of comparable studies.^8^, ^12^ However, the study has some limitations that warrant consideration. Its cross‐sectional design precludes causal inferences about the relationship between glymphatic dysfunction, sleep quality, and migraine frequency. Longitudinal studies are needed to determine whether improving sleep quality can directly enhance glymphatic function and reduce headache frequency. An additional limitation of the study is the absence of a control group of individuals without migraine; however, given that our outcome variable (monthly headache frequency) is not applicable to healthy individuals, a control group would not have meaningfully contributed to addressing our primary research questions. A further limitation is the subjective nature of sleep quality assessment that, although based on the widely used and validated PSQI, introduces inherent bias. More objective assessments of sleep quality such as polysomnography might have provided more objective data. We also recognized that various clinical and demographic confounders could influence the relationship under investigation. To address this, we ran a supplementary model that controlled not only for demographic variables (age and sex), but also for relevant clinical factors (years of disease history, medication overuse, cutaneous allodynia, aura status, and migraine type). The adjusted model confirmed the original results, thereby reinforcing the robustness of our findings. Nonetheless, we did not collect information on the duration of sleep disturbances if present and potential confounders such as socioeconomic status, psychological stress, and lifestyle habits (e.g., caffeine consumption and physical activity), which may influence both sleep quality and migraine frequency. Future research should aim to include them to further refine our understanding of these complex relationships.

Finally, given the exploratory nature of this study and the lack of available normative effect sizes for DTI‐ALPS in migraine populations—particularly in relation to sleep stratification—a formal sample size estimation was not feasible during the study design phase. Furthermore, current statistical tools do not provide validated methods to estimate power for NB regression models with interaction terms involving categorical moderators. To partly address this issue, we conducted a *post hoc* power analysis for the simple linear association between DTI‐ALPS and headache frequency within each sleep quality subgroup. In poor sleepers, the observed effect size (*f* ^2^ = 0.157) yielded a power of 92%, indicating that our sample was sufficient to detect this effect. In contrast, the association was weak in good sleepers (*f* ^2^ = 0.039), with a corresponding power of only 19%, a finding that aligns with our theoretical model, which does not expect a strong DTI‐ALPS–headache frequency relationship in this group.

Altogether, these results support the plausibility of the observed interaction and suggest that the relationship between impaired glymphatic function and migraine frequency may be especially pronounced in individuals with poor sleep quality. Nevertheless, we strongly encourage future studies to replicate these findings using prospectively powered designs and larger, independent samples, not only to validate our results but also to investigate potential age‐related variability in the proposed interaction.

## CONCLUSION

In conclusion, our findings suggested interplay between glymphatic dysfunction and sleep quality in the pathophysiology of migraine. The identification of sleep quality as a moderator of the relationship between glymphatic function and headache frequency paves the way for further research in the field, supporting the need of interventions targeting sleep disturbances in individuals with migraine to mitigate the relative burden, particularly those with evidence of glymphatic dysfunction.

## AUTHOR CONTRIBUTIONS

**Raffaele Ornello:** Conceptualization; formal analysis; methodology; writing – original draft. **Federico Salfi:** Conceptualization; formal analysis; methodology; writing – original draft. **Maria Grazia Vittorini:** Data curation. **Antonio Innocenzi:** Data curation. **Michele Ferrara:** Writing – review and editing. **Federico Bruno:** Data curation. **Francesca Pistoia:** Data curation; writing – review and editing. **Alessandra Splendiani:** Writing – review and editing. **Simona Sacco:** Conceptualization; data curation; supervision; writing – review and editing. **Federico De Santis:** Data curation.

## CONFLICT OF INTEREST STATEMENT

**Raffaele Ornello** reports personal fees or non‐financial support from AbbVie, Eli Lilly, Lundbeck, Novartis, Organon, Pfizer, and Teva; he is Editorial Board Member for The Journal of Headache and Pain and Confinia Cephalalgica, Associate Editor for Frontiers in Neurology and Arquivos de Neuropsiquiatria. **Simona Sacco** reports personal fees as speaker or advisor from Abbott, Allergan‐AbbVie, AstraZeneca, Bayer, Boheringer, Eli Lilly, Lundbeck, Pfizer, Teva, and research grants from Novartis and Uriach; she is president of the European Stroke Organization, Editor‐in‐Chief of Cephalalgia and Cephalalgia Reports, and Assistant Editor for Stroke. **Alessandra Splendiani** reports personal fees from Bayer and General Electric. **Federico Salfi**, **Maria Grazia Vittorini**, **Antonio Innocenzi**, **Federico De Santis**, **Federico Bruno**, **Francesca Pistoia**, and **Michele Ferrara** declare no conflicts of interest.

## Supporting information


Table S1.
