# Supplementary material for: The potential role of sleep quality in the relationship between glymphatic function and migraine frequency: Insights from a cross‐sectional study
Source: Headache. 2025 Aug 19;66(3):576–83. doi: 10.1111/head.15019 (PMC12951708; doi:10.1111/head.15019)
Supplement: Supplementary file 1 — Table S1. [file HEAD-66-576-s001.docx]

**Supplementary Table 1.** Results of the negative binomial model evaluating the moderating role of sleep quality on the relationship between DTI-ALPS index and monthly headache frequency (dependent variable) adjusting for demographic and clinically relevant variables.

|  | **Exp(B)** | **95 % CI** | **p-value** |
| --- | --- | --- | --- |
| (Intercept) | 6.42 | 0.72 – 57.03 | 0.10 |
| Age | 0.99 | 0.97 – 1.002 | 0.10 |
| Sex (female vs. male) | 0.96 | 0.70 – 1.32 | 0.82 |
| Years of disease history | 1.01 | 0.996 – 1.02 | 0.23 |
| Medication overuse (presence vs. absence) | 1.39 | 1.03 – 1.86 | **0.03** |
| Cutaneous allodynia (presence vs. absence) | 0.77 | 0.60 – 0.99 | **0.05** |
| Aura status (presence vs. absence) | 0.91 | 0.70 – 1.19 | 0.51 |
| Migraine diagnosis (chronic vs. episodic) | 2.23 | 1.68 – 2.95 | **< 0.001** |
| DTI-ALPS index | 1.40 | 0.42 – 4.66 | 0.58 |
| Sleep quality (poor vs. good) | 14.52 | 1.3 – 161.61 | **0.03** |
| DTI-ALPS index × Sleep quality | 0.20 | 0.05 – 0.89 | **0.03** |
